# *N*-Cyclo­dodecyl-5-(dimethyl­amino)­naphthalene-1-sulfonamide

**DOI:** 10.1107/S1600536808016164

**Published:** 2008-06-07

**Authors:** Conrad Fischer, Tobias Gruber, Wilhelm Seichter, Edwin Weber, Bakhtiyar T. Ibragimov

**Affiliations:** a Institut für Organische Chemie, TU Bergakademie Freiberg, Leipziger Strasse 29, D-09596 Freiberg/Sachsen, Germany; b Institute of Bioorganic Chemistry, Academy of Sciences of Uzbekistan, H Abdullaev 83, Tashkent 100125, Uzbekistan

## Abstract

The molecule of the title compound, C_24_H_36_N_2_O_2_S, displays a U-shaped conformation. The prominent inter­molecular inter­actions are N—H⋯O hydrogen bonds, resulting in the formation of dimers. Additional C—H⋯π contacts involving one of the methyl­ene groups of the macrocycle and the naphthalene rings of a neighbouring mol­ecule stabilize the packing structure. In the crystal structure, the cyclo­dodecyl ring is disordered over two positions; the site occupancy factors are *ca* 0.86 and 0.14.

## Related literature

For general background, see: Weber *et al.* (2004 ▶); Schönefeld *et al.* (2005 ▶); Gruber *et al.* (2008 ▶). For C—H⋯π contacts, see: Nishio (2004 ▶). For related structures, see: Dunitz & Shearer (1960 ▶); Rudert *et al.* (1994 ▶); Feiler *et al.* (1995 ▶).
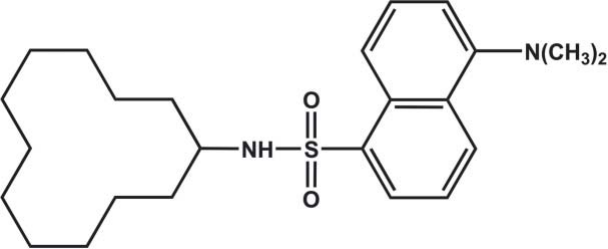


## Experimental

### 

#### Crystal data

C_24_H_36_N_2_O_2_S
*M* *_r_* = 416.62
Monoclinic, 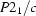

*a* = 10.3564 (3) Å
*b* = 13.5117 (4) Å
*c* = 16.2076 (4) Å
β = 95.814 (1)°
*V* = 2256.30 (11) Å^3^
*Z* = 4
Mo *K*α radiation
μ = 0.17 mm^−1^
*T* = 93 (2) K
0.54 × 0.42 × 0.29 mm

#### Data collection

Bruker Kappa APEXII CCD diffractometer
Absorption correction: multi scan (*SADABS*; Sheldrick, 2002 ▶) *T*_min_ = 0.826, *T*_max_ = 0.954
71103 measured reflections
14866 independent reflections
10604 reflections with *I* > 2σ(*I*)
*R*_int_ = 0.034

#### Refinement

*R*[*F* ^2^ > 2σ(*F* ^2^)] = 0.038
*wR*(*F* ^2^) = 0.120
*S* = 1.02
14866 reflections
469 parameters
H atoms treated by a mixture of independent and constrained refinement
Δρ_max_ = 0.46 e Å^−3^
Δρ_min_ = −0.26 e Å^−3^

### 

Data collection: *APEX2* (Bruker, 2004 ▶); cell refinement: *SAINT* (Bruker, 2004 ▶); data reduction: *SAINT*; program(s) used to solve structure: *SHELXTL* (Sheldrick, 2008 ▶); program(s) used to refine structure: *SHELXTL*; molecular graphics: *SHELXTL*; software used to prepare material for publication: *SHELXTL*.

## Supplementary Material

Crystal structure: contains datablocks global, I. DOI: 10.1107/S1600536808016164/xu2416sup1.cif

Structure factors: contains datablocks I. DOI: 10.1107/S1600536808016164/xu2416Isup2.hkl

Additional supplementary materials:  crystallographic information; 3D view; checkCIF report

## Figures and Tables

**Table 1:** Hydrogen-bond geometry (Å, °)

*D*—H⋯*A*	*D*—H	H⋯*A*	*D*⋯*A*	*D*—H⋯*A*
N2—H2⋯O1^i^	0.83 (1)	2.28 (1)	3.065 (1)	154 (1)
C4—H4⋯O1	0.95	2.34	3.026 (1)	128
C20—H20*A*⋯*CgA*^ii^	1.03 (1)	2.88 (1)	3.595 (1)	126 (1)
C20—H20*B*⋯*CgB*^ii^	0.96 (1)	2.84 (1)	3.613 (1)	140 (1)
C20*A*—H20*D*⋯*CgB*^ii^	0.99	2.81	3.792 (1)	170

Symmetry codes: (i) 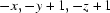; (ii) 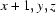. *CgA* and *CgB* are the centroids of the C1–C5/C10 and C5–C10 benzene rings, respectively.

**Fig. 1.**
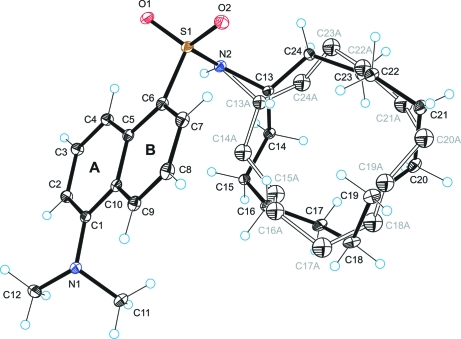
Molecular structure of the title compound with 30% probability displacement ellipsoids.

## supplementary crystallographic information

### Comment

The title compound (I) was prepared as part of our studies concerning fluorogenic receptor molecules with possible analytical applications (Weber *et al.*, 2004; Schönefeld *et al.*, 2005; Gruber *et al.*, 2008).

The molecular geometry of the molecule (figure 1) is best described by an angular conformation, whereas the cyclododecyl ring is disordered over two positions giving rise to an entirely filled cavity. The torsion angle are 71.44 (6)° (C6—S1—N2—C13) and 51.5 (3)° (C6—S1—N2—C13A), respectively, while the mean planes of the cyclododecylamine and the naphthalene include an angle of 20.1°. Weak C—H···π contacts (Nishio, 2004) involving one methylene group of the cycloalkyl ring and the two aromatic rings, and a C—H···O interaction between H4 and O1 stabilize the crystal packing. Furthermore, N—H···O hydrogen bonding leads to dimerization similar to carboxylic acids. The equatorial arrangement and conformation of the cyclododecyl moiety correspond to reported data (Dunitz & Shearer, 1960; Rudert *et al.*, 1994; Feiler *et al.*, 1995).

### Experimental

To a solution of cyclododecylamine (8.18 mmol) and triethylamine (14.39 mmol) in THF (50 ml), dansylchloride (9.63 mmol) in THF (50 ml) was added dropwise and refluxed for 2 h. After evaporation of the solvent under reduced pressure, the residue was dissolved in water/chloroform (100 ml of each). The organic extract was dried (Na_2_SO_4_) and the solvent distilled off. Recrystallization from *n*-hexane/dichloromethane (1:1) yielded light yellow crystals. (34%). Anal. Calcd. for C_24_H_36_N_2_O_2_S: C, 69.19; H, 8.71; N, 6.72; Found: C, 69.21; H, 8.83; N, 7.00%.

### Refinement

The cyclododecyl ring is disordered over two positions and refinement of occupancies converged to 0.8623:1377. H atoms of the major component of the disordered cyclododecyl were located in a difference Fourier map and refined isotropically. Imino H atom was also located in a difference Fourier map and refined isotropically. Other H atoms were positioned geometrically and allowed to ride on their parent atoms, with C–H = 0.95 - 0.99 Å, and refined in a riding mode with *U*_iso_(H) = 1.2 or 1.5*U*_eq_(C).

### Crystal data

**Table:**

C_24_H_36_N_2_O_2_S	*F*_000_ = 904
*M**_r_* = 416.62	*D*_x_ = 1.227 Mg m^−^^3^
Monoclinic, *P*2_1_/*c*	Melting point: 437 K
Hall symbol: -P 2ybc	Mo *K*α radiation λ = 0.71073 Å
*a* = 10.3564 (3) Å	Cell parameters from 9249 reflections
*b* = 13.5117 (4) Å	θ = 2.5–39.0º
*c* = 16.2076 (4) Å	µ = 0.17 mm^−^^1^
β = 95.8140 (10)º	*T* = 93 (2) K
*V* = 2256.30 (11) Å^3^	Plate, light yellow
*Z* = 4	0.54 × 0.42 × 0.29 mm

### Data collection

**Table:**

Bruker Kappa APEXII CCD diffractometer	14866 independent reflections
Radiation source: fine-focus sealed tube	10604 reflections with *I* > 2σ(*I*)
Monochromator: graphite	*R*_int_ = 0.034
*T* = 93(2) K	θ_max_ = 41.0º
φ and ω scans	θ_min_ = 2.0º
Absorption correction: multi scan(SADABS; Sheldrick, 2002)	*h* = −18→19
*T*_min_ = 0.826, *T*_max_ = 0.954	*k* = −24→24
71103 measured reflections	*l* = −29→29

### Refinement

**Table:**

Refinement on *F*^2^	Secondary atom site location: difference Fourier map
Least-squares matrix: full	Hydrogen site location: inferred from neighbouring sites
*R*[*F*^2^ > 2σ(*F*^2^)] = 0.039	H atoms treated by a mixture of independent and constrained refinement
*wR*(*F*^2^) = 0.120	*w* = 1/[σ^2^(*F*_o_^2^) + (0.0655*P*)^2^ + 0.1633*P*] where *P* = (*F*_o_^2^ + 2*F*_c_^2^)/3
*S* = 1.02	(Δ/σ)_max_ < 0.001
14866 reflections	Δρ_max_ = 0.46 e Å^−^^3^
469 parameters	Δρ_min_ = −0.26 e Å^−^^3^
Primary atom site location: structure-invariant direct methods	Extinction correction: none

### Special details

**Table:**

Experimental. ^1^H NMR (400 MHz, CDCl_3_, δ, p.p.m.): 0.95 (4*H*, m, CH_2_, 1.09 (4*H*, m, CH_2_), 1.20 (12*H*, m, CH_2_), 1.41 (2*H*, m, CH_2_), 2.88 (6*H*, s, N(CH_3_)_2_), 3.20 (1*H*, m, C*H*NH), 4.57 (1*H*, s, CHN*H*), 7.17 (1*H*, d, Ar*H*), 7.53 (2*H*, d, ArH), 8.32 (2*H*, m, ArH), 8.52 (1*H*, d, ArH). ^13^C NMR (100 MHz, CDCl_3_, δ, p.p.m.): 21.09, 23.06, 23.17, 23.24, 23.52, 31.13 (*C*H_2_), 45.36 (N(*C*H_3_)_2_), 50.53 (*C*HNH), 115.03 (6-Ar*C*), 119.00, 123.09 (2-, 8-Ar*C*), 128.21, 129.61, 129.76, 129.84, 130.24 (3-,4-,4a-,7-,8a-Ar*C*), 135.57 (1-Ar*C*), 151.89 (5-Ar*C*). IR (KBr): 3290 (N—H); 3076, 3051 (C—H_ar_); 2937, 2862 (C—H_alk_); 2787 (N—CH_3_); 1620, 1592, 1574 (C?C); 1474; 1453; 1413; 1356; 1313; 1231; 1206; 1164; 1150; 1092; 1074; 1060; 1028; 946; 882; 792; 689; 632; 578.
Geometry. All e.s.d.'s (except the e.s.d. in the dihedral angle between two l.s. planes) are estimated using the full covariance matrix. The cell e.s.d.'s are taken into account individually in the estimation of e.s.d.'s in distances, angles and torsion angles; correlations between e.s.d.'s in cell parameters are only used when they are defined by crystal symmetry. An approximate (isotropic) treatment of cell e.s.d.'s is used for estimating e.s.d.'s involving l.s. planes.
Refinement. Refinement of *F*^2^ against ALL reflections. The weighted *R*-factor *wR* and goodness of fit *S* are based on *F*^2^, conventional *R*-factors *R* are based on *F*, with *F* set to zero for negative *F*^2^. The threshold expression of *F*^2^ > σ(*F*^2^) is used only for calculating *R*-factors(gt) *etc*. and is not relevant to the choice of reflections for refinement. *R*-factors based on *F*^2^ are statistically about twice as large as those based on *F*, and *R*- factors based on ALL data will be even larger.

### Fractional atomic coordinates and isotropic or equivalent isotropic displacement parameters (Å^2^)

**Table:**

	*x*	*y*	*z*	*U*_iso_*/*U*_eq_	Occ. (<1)
S1	0.039201 (15)	0.332136 (11)	0.456193 (9)	0.01773 (4)	
O1	−0.06052 (5)	0.36676 (4)	0.50517 (3)	0.02297 (10)	
O2	0.11146 (5)	0.24527 (4)	0.48235 (3)	0.02405 (10)	
N1	−0.23380 (5)	0.42906 (4)	0.08519 (3)	0.01851 (9)	
N2	0.14083 (5)	0.42236 (4)	0.45239 (3)	0.01769 (9)	
H2'	0.1060 (11)	0.4782 (9)	0.4485 (7)	0.034 (3)*	
C1	−0.21013 (5)	0.44354 (4)	0.17181 (4)	0.01559 (9)	
C2	−0.25882 (6)	0.52375 (5)	0.21154 (4)	0.01812 (10)	
H2	−0.3110	0.5712	0.1804	0.022*	
C3	−0.23173 (7)	0.53578 (5)	0.29785 (4)	0.01954 (11)	
H3	−0.2671	0.5909	0.3242	0.023*	
C4	−0.15557 (6)	0.46971 (5)	0.34466 (4)	0.01791 (10)	
H4	−0.1360	0.4807	0.4025	0.021*	
C5	−0.10584 (6)	0.38498 (4)	0.30710 (4)	0.01552 (9)	
C6	−0.02721 (6)	0.31162 (4)	0.35209 (4)	0.01712 (10)	
C7	0.01112 (7)	0.22697 (5)	0.31416 (4)	0.02077 (11)	
H7	0.0636	0.1796	0.3452	0.025*	
C8	−0.02742 (7)	0.21044 (5)	0.22930 (5)	0.02228 (12)	
H8	−0.0040	0.1506	0.2039	0.027*	
C9	−0.09844 (6)	0.28033 (5)	0.18340 (4)	0.01948 (10)	
H9	−0.1227	0.2687	0.1261	0.023*	
C10	−0.13641 (5)	0.36992 (4)	0.21999 (4)	0.01529 (9)	
C11	−0.11742 (7)	0.43858 (6)	0.04101 (4)	0.02615 (13)	
H11A	−0.0945	0.5087	0.0370	0.039*	
H11B	−0.1349	0.4107	−0.0148	0.039*	
H11C	−0.0453	0.4027	0.0714	0.039*	
C12	−0.33772 (7)	0.48886 (6)	0.04336 (4)	0.02499 (13)	
H12A	−0.4145	0.4848	0.0739	0.037*	
H12B	−0.3593	0.4641	−0.0132	0.037*	
H12C	−0.3092	0.5579	0.0413	0.037*	
C13	0.26599 (10)	0.40723 (8)	0.41631 (6)	0.01599 (15)	0.8623 (12)
H13	0.2564 (11)	0.3489 (8)	0.3830 (7)	0.015 (2)*	0.8623 (12)
C14	0.29031 (8)	0.49533 (6)	0.36018 (5)	0.01951 (13)	0.8623 (12)
H14A	0.3902 (12)	0.5005 (9)	0.3563 (7)	0.026 (3)*	0.8623 (12)
H14B	0.2651 (13)	0.5557 (10)	0.3868 (8)	0.031 (3)*	0.8623 (12)
C15	0.21831 (8)	0.48964 (6)	0.27273 (5)	0.02269 (15)	0.8623 (12)
H15A	0.1238 (12)	0.4829 (9)	0.2768 (7)	0.025 (3)*	0.8623 (12)
H15B	0.2324 (13)	0.5552 (10)	0.2449 (8)	0.031 (3)*	0.8623 (12)
C16	0.25964 (11)	0.40350 (9)	0.21972 (7)	0.02426 (19)	0.8623 (12)
H16A	0.2467 (12)	0.3403 (9)	0.2514 (8)	0.020 (3)*	0.8623 (12)
H16B	0.2029 (13)	0.4015 (10)	0.1665 (8)	0.032 (3)*	0.8623 (12)
C17	0.39987 (9)	0.40970 (6)	0.19824 (5)	0.02443 (15)	0.8623 (12)
H17A	0.4599 (12)	0.4308 (9)	0.2472 (8)	0.026 (3)*	0.8623 (12)
H17B	0.4033 (14)	0.4678 (11)	0.1564 (9)	0.041 (4)*	0.8623 (12)
C18	0.45099 (12)	0.31286 (7)	0.16479 (6)	0.02798 (18)	0.8623 (12)
H18A	0.5359 (15)	0.3266 (11)	0.1369 (9)	0.040 (4)*	0.8623 (12)
H18B	0.3903 (16)	0.2865 (13)	0.1204 (10)	0.051 (4)*	0.8623 (12)
C19	0.47808 (14)	0.23333 (9)	0.23171 (8)	0.0257 (2)	0.8623 (12)
H19A	0.4022 (12)	0.2275 (9)	0.2587 (8)	0.027 (3)*	0.8623 (12)
H19B	0.4843 (15)	0.1656 (11)	0.2067 (9)	0.042 (4)*	0.8623 (12)
C20	0.59746 (8)	0.25578 (6)	0.29262 (6)	0.02371 (15)	0.8623 (12)
H20A	0.6066 (13)	0.3314 (9)	0.2995 (8)	0.027 (3)*	0.8623 (12)
H20B	0.6733 (13)	0.2330 (10)	0.2690 (8)	0.033 (3)*	0.8623 (12)
C21	0.59636 (9)	0.20577 (6)	0.37710 (6)	0.02530 (16)	0.8623 (12)
H21A	0.5887 (13)	0.1346 (10)	0.3676 (8)	0.032 (3)*	0.8623 (12)
H21B	0.6813 (12)	0.2197 (9)	0.4102 (8)	0.025 (3)*	0.8623 (12)
C22	0.48776 (10)	0.24042 (7)	0.42801 (6)	0.02177 (15)	0.8623 (12)
H22A	0.4013 (12)	0.2295 (9)	0.3935 (7)	0.024 (3)*	0.8623 (12)
H22B	0.4854 (13)	0.1965 (10)	0.4788 (8)	0.030 (3)*	0.8623 (12)
C23	0.50024 (8)	0.34853 (6)	0.45558 (5)	0.02195 (14)	0.8623 (12)
H23A	0.5261 (11)	0.3903 (9)	0.4092 (7)	0.022 (3)*	0.8623 (12)
H23B	0.5757 (12)	0.3529 (9)	0.4994 (8)	0.027 (3)*	0.8623 (12)
C24	0.37593 (8)	0.39027 (6)	0.48608 (5)	0.01908 (13)	0.8623 (12)
H24A	0.3437 (12)	0.3419 (8)	0.5269 (8)	0.021 (3)*	0.8623 (12)
H24B	0.3937 (12)	0.4520 (9)	0.5149 (8)	0.026 (3)*	0.8623 (12)
C13A	0.2423 (7)	0.4184 (5)	0.3938 (4)	0.0194 (11)	0.1377 (12)
H13A	0.2460	0.3492	0.3722	0.023*	0.1377 (12)
C14A	0.2135 (5)	0.4874 (4)	0.3205 (3)	0.0217 (9)	0.1377 (12)
H14C	0.2274	0.5566	0.3396	0.026*	0.1377 (12)
H14D	0.1211	0.4805	0.2989	0.026*	0.1377 (12)
C15A	0.2983 (6)	0.4673 (4)	0.2497 (4)	0.0269 (10)	0.1377 (12)
H15C	0.2839	0.5208	0.2081	0.032*	0.1377 (12)
H15D	0.3907	0.4697	0.2724	0.032*	0.1377 (12)
C16A	0.2720 (8)	0.3687 (6)	0.2067 (5)	0.0289 (13)	0.1377 (12)
H16C	0.1888	0.3727	0.1710	0.035*	0.1377 (12)
H16D	0.2629	0.3171	0.2491	0.035*	0.1377 (12)
C17A	0.3806 (6)	0.3380 (5)	0.1532 (4)	0.0292 (11)	0.1377 (12)
H17C	0.3500	0.2805	0.1186	0.035*	0.1377 (12)
H17D	0.3959	0.3932	0.1152	0.035*	0.1377 (12)
C18A	0.5089 (6)	0.3109 (4)	0.2021 (4)	0.0262 (10)	0.1377 (12)
H18C	0.5739	0.3006	0.1621	0.031*	0.1377 (12)
H18D	0.5380	0.3684	0.2370	0.031*	0.1377 (12)
C19A	0.5103 (9)	0.2219 (7)	0.2568 (5)	0.0290 (14)	0.1377 (12)
H19C	0.5224	0.1629	0.2222	0.035*	0.1377 (12)
H19D	0.4235	0.2160	0.2770	0.035*	0.1377 (12)
C20A	0.6097 (5)	0.2175 (4)	0.3309 (3)	0.0226 (9)	0.1377 (12)
H20C	0.6965	0.2290	0.3121	0.027*	0.1377 (12)
H20D	0.6094	0.1500	0.3546	0.027*	0.1377 (12)
C21A	0.5887 (5)	0.2918 (4)	0.3989 (3)	0.0226 (9)	0.1377 (12)
H21C	0.6677	0.2934	0.4388	0.027*	0.1377 (12)
H21D	0.5779	0.3584	0.3736	0.027*	0.1377 (12)
C22A	0.4735 (6)	0.2708 (5)	0.4459 (4)	0.0227 (10)	0.1377 (12)
H22C	0.4006	0.2494	0.4056	0.027*	0.1377 (12)
H22D	0.4958	0.2145	0.4837	0.027*	0.1377 (12)
C23A	0.4269 (5)	0.3538 (4)	0.4958 (3)	0.0227 (9)	0.1377 (12)
H23C	0.5009	0.3787	0.5335	0.027*	0.1377 (12)
H23D	0.3614	0.3277	0.5305	0.027*	0.1377 (12)
C24A	0.3680 (5)	0.4397 (4)	0.4452 (3)	0.0220 (9)	0.1377 (12)
H24C	0.3535	0.4948	0.4835	0.026*	0.1377 (12)
H24D	0.4317	0.4628	0.4078	0.026*	0.1377 (12)

### Atomic displacement parameters (Å^2^)

**Table:**

	*U*^11^	*U*^22^	*U*^33^	*U*^12^	*U*^13^	*U*^23^
S1	0.01813 (7)	0.01768 (7)	0.01798 (6)	0.00164 (5)	0.00484 (5)	0.00483 (5)
O1	0.0224 (2)	0.0277 (2)	0.0202 (2)	0.00305 (18)	0.00882 (17)	0.00475 (17)
O2	0.0248 (2)	0.0197 (2)	0.0278 (2)	0.00326 (17)	0.00366 (18)	0.00930 (18)
N1	0.0155 (2)	0.0247 (2)	0.01568 (19)	0.00039 (17)	0.00316 (16)	−0.00216 (17)
N2	0.0186 (2)	0.0165 (2)	0.0185 (2)	0.00146 (17)	0.00489 (17)	0.00115 (16)
C1	0.0131 (2)	0.0173 (2)	0.0167 (2)	−0.00111 (17)	0.00304 (17)	−0.00180 (17)
C2	0.0187 (2)	0.0171 (2)	0.0185 (2)	0.00267 (19)	0.00184 (19)	−0.00127 (19)
C3	0.0226 (3)	0.0173 (2)	0.0189 (2)	0.0045 (2)	0.0028 (2)	−0.00262 (19)
C4	0.0204 (2)	0.0166 (2)	0.0170 (2)	0.00290 (19)	0.00259 (19)	−0.00208 (18)
C5	0.0148 (2)	0.0140 (2)	0.0182 (2)	−0.00004 (17)	0.00383 (17)	−0.00080 (17)
C6	0.0167 (2)	0.0147 (2)	0.0205 (2)	0.00090 (18)	0.00426 (18)	0.00131 (18)
C7	0.0205 (3)	0.0149 (2)	0.0275 (3)	0.00235 (19)	0.0049 (2)	0.0006 (2)
C8	0.0221 (3)	0.0164 (2)	0.0290 (3)	0.0023 (2)	0.0058 (2)	−0.0049 (2)
C9	0.0182 (2)	0.0176 (2)	0.0231 (3)	−0.00017 (19)	0.0049 (2)	−0.0059 (2)
C10	0.0136 (2)	0.0147 (2)	0.0181 (2)	−0.00075 (16)	0.00396 (17)	−0.00231 (17)
C11	0.0223 (3)	0.0366 (4)	0.0209 (3)	0.0001 (3)	0.0090 (2)	−0.0012 (3)
C12	0.0225 (3)	0.0328 (3)	0.0192 (3)	0.0043 (2)	−0.0004 (2)	−0.0007 (2)
C13	0.0180 (4)	0.0155 (3)	0.0149 (4)	0.0001 (3)	0.0041 (3)	−0.0004 (3)
C14	0.0211 (3)	0.0178 (3)	0.0202 (3)	−0.0010 (2)	0.0051 (2)	0.0013 (2)
C15	0.0194 (3)	0.0273 (4)	0.0217 (3)	0.0012 (3)	0.0042 (2)	0.0074 (3)
C16	0.0239 (4)	0.0321 (5)	0.0170 (4)	−0.0055 (4)	0.0032 (3)	0.0019 (4)
C17	0.0291 (4)	0.0230 (3)	0.0230 (3)	−0.0026 (3)	0.0113 (3)	0.0045 (3)
C18	0.0400 (6)	0.0270 (4)	0.0185 (3)	−0.0025 (4)	0.0104 (4)	−0.0012 (3)
C19	0.0334 (6)	0.0189 (4)	0.0258 (5)	−0.0070 (4)	0.0072 (4)	−0.0033 (4)
C20	0.0234 (3)	0.0205 (3)	0.0289 (4)	−0.0014 (3)	0.0111 (3)	−0.0005 (3)
C21	0.0275 (4)	0.0203 (3)	0.0292 (4)	0.0075 (3)	0.0080 (3)	0.0018 (3)
C22	0.0267 (4)	0.0159 (3)	0.0237 (4)	0.0017 (3)	0.0076 (3)	0.0011 (3)
C23	0.0187 (3)	0.0197 (3)	0.0271 (3)	0.0014 (2)	0.0011 (3)	−0.0044 (3)
C24	0.0201 (3)	0.0196 (3)	0.0172 (3)	0.0015 (3)	0.0002 (2)	−0.0037 (2)
C13A	0.018 (3)	0.020 (2)	0.020 (3)	0.0042 (18)	0.002 (2)	0.002 (2)
C14A	0.027 (2)	0.0186 (19)	0.020 (2)	0.0021 (16)	0.0067 (16)	0.0068 (15)
C15A	0.029 (2)	0.023 (2)	0.031 (2)	0.0003 (18)	0.012 (2)	0.0089 (18)
C16A	0.023 (3)	0.036 (4)	0.028 (3)	−0.007 (3)	0.002 (2)	0.001 (3)
C17A	0.029 (3)	0.040 (3)	0.020 (2)	−0.002 (2)	0.0107 (19)	0.007 (2)
C18A	0.030 (3)	0.026 (2)	0.024 (2)	−0.0026 (19)	0.012 (2)	0.0017 (18)
C19A	0.033 (4)	0.028 (3)	0.027 (3)	−0.007 (3)	0.011 (3)	−0.006 (3)
C20A	0.027 (2)	0.0194 (19)	0.022 (2)	0.0026 (16)	0.0073 (17)	0.0040 (17)
C21A	0.0190 (19)	0.024 (2)	0.025 (2)	0.0020 (16)	0.0036 (16)	−0.0023 (17)
C22A	0.024 (2)	0.021 (2)	0.023 (2)	0.0085 (19)	0.0006 (18)	0.0006 (19)
C23A	0.0167 (19)	0.026 (2)	0.024 (2)	0.0079 (17)	−0.0056 (15)	−0.0060 (17)
C24A	0.0172 (18)	0.0193 (19)	0.029 (2)	0.0022 (15)	−0.0011 (16)	−0.0050 (17)

### Geometric parameters (Å, °)

**Table:**

S1—O2	1.4328 (5)	C19—H19A	0.941 (13)
S1—O1	1.4428 (5)	C19—H19B	1.006 (15)
S1—N2	1.6157 (6)	C20—C21	1.5280 (13)
S1—C6	1.7781 (7)	C20—H20A	1.031 (12)
N1—C1	1.4140 (8)	C20—H20B	0.959 (14)
N1—C12	1.4570 (9)	C21—C22	1.5340 (13)
N1—C11	1.4687 (8)	C21—H21A	0.975 (13)
N2—C13A	1.487 (7)	C21—H21B	1.001 (12)
N2—C13	1.4891 (12)	C22—C23	1.5292 (13)
N2—H2'	0.835 (12)	C22—H22A	1.017 (12)
C1—C2	1.3818 (8)	C22—H22B	1.017 (13)
C1—C10	1.4357 (9)	C23—C24	1.5326 (11)
C2—C3	1.4080 (9)	C23—H23A	0.997 (12)
C2—H2	0.9500	C23—H23B	1.003 (13)
C3—C4	1.3686 (9)	C24—H24A	1.011 (12)
C3—H3	0.9500	C24—H24B	0.965 (13)
C4—C5	1.4178 (8)	C13A—C24A	1.501 (8)
C4—H4	0.9500	C13A—C14A	1.516 (8)
C5—C10	1.4298 (8)	C13A—H13	0.968 (13)
C5—C6	1.4341 (8)	C13A—H13A	1.0000
C6—C7	1.3760 (9)	C14A—C15A	1.538 (7)
C7—C8	1.4105 (10)	C14A—H14C	0.9900
C7—H7	0.9500	C14A—H14D	0.9900
C8—C9	1.3694 (10)	C15A—C16A	1.516 (10)
C8—H8	0.9500	C15A—H15C	0.9900
C9—C10	1.4209 (8)	C15A—H15D	0.9900
C9—H9	0.9500	C16A—C17A	1.545 (10)
C11—H11A	0.9800	C16A—H16C	0.9900
C11—H11B	0.9800	C16A—H16D	0.9900
C11—H11C	0.9800	C17A—C18A	1.521 (9)
C12—H12A	0.9800	C17A—H17C	0.9900
C12—H12B	0.9800	C17A—H17D	0.9900
C12—H12C	0.9800	C18A—C19A	1.494 (10)
C13—C14	1.5344 (12)	C18A—H18C	0.9900
C13—C24	1.5384 (13)	C18A—H18D	0.9900
C13—H13	0.954 (11)	C19A—C20A	1.501 (10)
C14—C15	1.5352 (12)	C19A—H19C	0.9900
C14—H14A	1.045 (12)	C19A—H19D	0.9900
C14—H14B	0.970 (13)	C20A—C21A	1.523 (7)
C15—C16	1.5331 (15)	C20A—H20C	0.9900
C15—H15A	0.991 (12)	C20A—H20D	0.9900
C15—H15B	1.012 (13)	C21A—C22A	1.507 (8)
C16—C17	1.5296 (14)	C21A—H21C	0.9900
C16—H16A	1.012 (12)	C21A—H21D	0.9900
C16—H16B	0.994 (13)	C22A—C23A	1.490 (8)
C17—C18	1.5312 (13)	C22A—H22C	0.9900
C17—H17A	0.998 (13)	C22A—H22D	0.9900
C17—H17B	1.040 (15)	C23A—C24A	1.513 (7)
C18—C19	1.5324 (16)	C23A—H23C	0.9900
C18—H18A	1.045 (15)	C23A—H23D	0.9900
C18—H18B	0.974 (17)	C24A—H24C	0.9900
C19—C20	1.5323 (16)	C24A—H24D	0.9900
			
O2—S1—O1	118.96 (3)	C22—C21—H21B	107.9 (7)
O2—S1—N2	107.86 (3)	H21A—C21—H21B	108.9 (11)
O1—S1—N2	106.36 (3)	C23—C22—C21	113.82 (7)
O2—S1—C6	107.06 (3)	C23—C22—H22A	110.1 (7)
O1—S1—C6	110.06 (3)	C21—C22—H22A	108.2 (7)
N2—S1—C6	105.80 (3)	C23—C22—H22B	109.2 (7)
C1—N1—C12	115.52 (5)	C21—C22—H22B	109.6 (8)
C1—N1—C11	113.81 (5)	H22A—C22—H22B	105.6 (10)
C12—N1—C11	109.18 (6)	C22—C23—C24	113.38 (7)
C13A—N2—S1	120.1 (3)	C22—C23—H23A	110.0 (7)
C13—N2—S1	120.51 (5)	C24—C23—H23A	109.5 (7)
C13A—N2—H2'	108.1 (8)	C22—C23—H23B	107.4 (7)
C13—N2—H2'	118.7 (8)	C24—C23—H23B	111.7 (7)
S1—N2—H2'	114.0 (8)	H23A—C23—H23B	104.5 (10)
C2—C1—N1	122.44 (6)	C23—C24—C13	113.65 (7)
C2—C1—C10	119.27 (5)	C23—C24—H24A	108.5 (7)
N1—C1—C10	118.25 (5)	C13—C24—H24A	108.0 (7)
C1—C2—C3	120.54 (6)	C23—C24—H24B	110.5 (7)
C1—C2—H2	119.7	C13—C24—H24B	108.5 (7)
C3—C2—H2	119.7	H24A—C24—H24B	107.6 (10)
C4—C3—C2	121.40 (6)	N2—C13A—C24A	105.5 (5)
C4—C3—H3	119.3	N2—C13A—C14A	112.5 (5)
C2—C3—H3	119.3	C24A—C13A—C14A	114.0 (6)
C3—C4—C5	120.18 (6)	C24A—C13A—H13	98.5 (9)
C3—C4—H4	119.9	N2—C13A—H13A	108.2
C5—C4—H4	119.9	C13A—C14A—C15A	113.2 (5)
C4—C5—C10	119.02 (5)	C13A—C14A—H14C	108.9
C4—C5—C6	123.51 (5)	C15A—C14A—H14C	108.9
C10—C5—C6	117.47 (5)	H15A—C14A—H14C	109.7
C7—C6—C5	121.47 (6)	C13A—C14A—H14D	108.9
C7—C6—S1	116.88 (5)	C15A—C14A—H14D	108.9
C5—C6—S1	121.32 (4)	H14C—C14A—H14D	107.8
C6—C7—C8	120.09 (6)	C16A—C15A—C14A	114.1 (5)
C6—C7—H7	120.0	C16A—C15A—H15C	108.7
C8—C7—H7	120.0	C14A—C15A—H15C	108.7
C9—C8—C7	120.25 (6)	C16A—C15A—H15D	108.7
C9—C8—H8	119.9	C14A—C15A—H15D	108.7
C7—C8—H8	119.9	H15C—C15A—H15D	107.6
C8—C9—C10	121.13 (6)	C15A—C16A—C17A	112.8 (6)
C8—C9—H9	119.4	C15A—C16A—H16C	109.0
C10—C9—H9	119.4	C17A—C16A—H16C	109.0
C9—C10—C5	119.36 (6)	C15A—C16A—H16D	109.0
C9—C10—C1	121.17 (5)	C17A—C16A—H16D	109.0
C5—C10—C1	119.40 (5)	H16C—C16A—H16D	107.8
N2—C13—C14	109.15 (7)	C18A—C17A—C16A	114.7 (5)
N2—C13—C24	109.92 (7)	C18A—C17A—H17C	108.6
C14—C13—C24	113.43 (8)	C16A—C17A—H17C	108.6
N2—C13—H13	106.9 (7)	C18A—C17A—H17D	108.6
C14—C13—H13	108.6 (7)	C16A—C17A—H17D	108.6
C24—C13—H13	108.6 (7)	H17C—C17A—H17D	107.6
C13—C14—C15	114.52 (7)	C19A—C18A—H18C	108.0
C13—C14—H14A	108.1 (7)	C17A—C18A—H18C	108.0
C15—C14—H14A	109.8 (7)	C19A—C18A—H18D	108.0
C13—C14—H14B	108.7 (8)	C17A—C18A—H18D	108.0
C15—C14—H14B	109.0 (8)	H18C—C18A—H18D	107.2
H14A—C14—H14B	106.4 (10)	C18A—C19A—C20A	118.1 (7)
C16—C15—C14	114.73 (7)	C18A—C19A—H19C	107.8
C16—C15—H15A	107.5 (7)	C20A—C19A—H19C	107.8
C14—C15—H15A	109.5 (7)	C18A—C19A—H19D	107.8
C16—C15—H15B	110.7 (7)	C20A—C19A—H19D	107.8
C14—C15—H15B	106.8 (7)	H19C—C19A—H19D	107.1
H15A—C15—H15B	107.3 (10)	C19A—C20A—C21A	114.6 (5)
C17—C16—C15	114.29 (9)	C19A—C20A—H20C	108.6
C17—C16—H16A	109.8 (7)	C21A—C20A—H20C	108.6
C15—C16—H16A	107.3 (7)	C19A—C20A—H20D	108.6
C17—C16—H16B	107.1 (8)	C21A—C20A—H20D	108.6
C15—C16—H16B	109.5 (8)	H20C—C20A—H20D	107.6
H16A—C16—H16B	108.7 (10)	C22A—C21A—C20A	114.9 (5)
C16—C17—C18	113.83 (8)	C22A—C21A—H21C	108.6
C16—C17—H17A	111.3 (7)	C20A—C21A—H21C	108.6
C18—C17—H17A	108.4 (7)	C22A—C21A—H21D	108.6
C16—C17—H17B	106.6 (8)	C20A—C21A—H21D	108.6
C18—C17—H17B	112.2 (8)	H21C—C21A—H21D	107.5
H17A—C17—H17B	104.0 (11)	C23A—C22A—C21A	116.7 (5)
C17—C18—C19	113.18 (8)	C23A—C22A—H22B	115.8 (9)
C17—C18—H18A	109.7 (8)	C21A—C22A—H22B	110.4 (9)
C19—C18—H18A	109.2 (8)	C23A—C22A—H22C	108.1
C17—C18—H18B	110.7 (10)	C21A—C22A—H22C	108.1
C19—C18—H18B	108.9 (10)	C23A—C22A—H22D	108.1
H18A—C18—H18B	104.8 (13)	C21A—C22A—H22D	108.1
C20—C19—C18	113.27 (9)	H22C—C22A—H22D	107.3
C20—C19—H19A	112.0 (8)	C24A—C23A—H23B	112.0 (6)
C18—C19—H19A	106.7 (8)	C24A—C23A—H24A	94.1 (8)
C20—C19—H19B	111.1 (9)	C24A—C23A—H24B	45.9 (5)
C18—C19—H19B	111.6 (9)	C22A—C23A—H23C	108.6
H19A—C19—H19B	101.6 (11)	C24A—C23A—H23C	108.6
C21—C20—C19	114.27 (8)	C22A—C23A—H23D	108.6
C21—C20—H20A	110.5 (7)	C24A—C23A—H23D	108.6
C19—C20—H20A	109.0 (7)	H23C—C23A—H23D	107.6
C21—C20—H20B	107.2 (8)	C13A—C24A—C23A	115.7 (5)
C19—C20—H20B	108.6 (8)	C13A—C24A—H24C	108.4
H20A—C20—H20B	107.0 (11)	C23A—C24A—H24C	108.4
C20—C21—C22	114.80 (7)	C13A—C24A—H24D	108.4
C20—C21—H21A	107.6 (8)	C23A—C24A—H24D	108.4
C22—C21—H21A	109.4 (8)	H24C—C24A—H24D	107.4
C20—C21—H21B	108.1 (7)		
			
O2—S1—N2—C13A	62.8 (3)	C2—C1—C10—C5	−5.06 (8)
O1—S1—N2—C13A	−168.5 (3)	N1—C1—C10—C5	177.07 (5)
C6—S1—N2—C13A	−51.5 (3)	C13A—N2—C13—C14	42.4 (10)
O2—S1—N2—C13	42.85 (6)	S1—N2—C13—C14	136.27 (6)
O1—S1—N2—C13	171.52 (6)	C13A—N2—C13—C24	167.4 (11)
C6—S1—N2—C13	−71.44 (6)	S1—N2—C13—C24	−98.73 (7)
C12—N1—C1—C2	−14.76 (9)	N2—C13—C14—C15	−81.47 (9)
C11—N1—C1—C2	112.73 (7)	C24—C13—C14—C15	155.60 (7)
C12—N1—C1—C10	163.04 (6)	C13—C14—C15—C16	−64.30 (10)
C11—N1—C1—C10	−69.47 (7)	C14—C15—C16—C17	−65.13 (10)
N1—C1—C2—C3	−179.36 (6)	C15—C16—C17—C18	165.48 (8)
C10—C1—C2—C3	2.87 (9)	C16—C17—C18—C19	−72.80 (12)
C1—C2—C3—C4	0.86 (10)	C17—C18—C19—C20	−71.53 (13)
C2—C3—C4—C5	−2.33 (10)	C18—C19—C20—C21	155.92 (8)
C3—C4—C5—C10	0.04 (9)	C19—C20—C21—C22	−64.74 (11)
C3—C4—C5—C6	−178.96 (6)	C20—C21—C22—C23	−65.97 (11)
C4—C5—C6—C7	175.23 (6)	C21—C22—C23—C24	165.03 (7)
C10—C5—C6—C7	−3.79 (9)	C22—C23—C24—C13	−71.56 (10)
C4—C5—C6—S1	−11.56 (8)	N2—C13—C24—C23	165.44 (7)
C10—C5—C6—S1	169.42 (4)	C14—C13—C24—C23	−72.05 (9)
O2—S1—C6—C7	−6.61 (6)	C13—N2—C13A—C24A	−32.4 (8)
O1—S1—C6—C7	−137.25 (5)	S1—N2—C13A—C24A	−128.7 (4)
N2—S1—C6—C7	108.23 (5)	C13—N2—C13A—C14A	−157.3 (15)
O2—S1—C6—C5	179.88 (5)	S1—N2—C13A—C14A	106.4 (5)
O1—S1—C6—C5	49.24 (6)	N2—C13A—C14A—C15A	−166.6 (5)
N2—S1—C6—C5	−65.28 (5)	C24A—C13A—C14A—C15A	73.3 (7)
C5—C6—C7—C8	−0.35 (10)	C13A—C14A—C15A—C16A	66.8 (7)
S1—C6—C7—C8	−173.85 (5)	C14A—C15A—C16A—C17A	−164.6 (5)
C6—C7—C8—C9	2.78 (10)	C15A—C16A—C17A—C18A	69.2 (8)
C7—C8—C9—C10	−0.90 (10)	C16A—C17A—C18A—C19A	64.7 (8)
C8—C9—C10—C5	−3.35 (9)	C17A—C18A—C19A—C20A	−153.6 (6)
C8—C9—C10—C1	179.58 (6)	C18A—C19A—C20A—C21A	68.7 (8)
C4—C5—C10—C9	−173.51 (6)	C19A—C20A—C21A—C22A	70.4 (7)
C6—C5—C10—C9	5.55 (8)	C20A—C21A—C22A—C23A	−163.5 (5)
C4—C5—C10—C1	3.62 (8)	C21A—C22A—C23A—C24A	67.6 (6)
C6—C5—C10—C1	−177.32 (5)	N2—C13A—C24A—C23A	79.3 (6)
C2—C1—C10—C9	172.01 (6)	C14A—C13A—C24A—C23A	−156.7 (5)
N1—C1—C10—C9	−5.86 (8)	C22A—C23A—C24A—C13A	67.2 (7)

### Hydrogen-bond geometry (Å, °)

**Table:**

*D*—H···*A*	*D*—H	H···*A*	*D*···*A*	*D*—H···*A*
N2—H2···O1^i^	0.84 (1)	2.28 (1)	3.065 (1)	154 (1)
C4—H4···O1	0.95	2.34	3.026 (1)	128
C20—H20A···*Cg*A^ii^	1.031 (12)	2.88 (1)	3.595 (1)	126 (1)
C20—H20B···*Cg*B^ii^	0.959 (14)	2.84 (1)	3.613 (1)	140 (1)
C20A—H20D···*Cg*B^ii^	0.99	2.81	3.792 (1)	170

Symmetry codes: (i) −*x*, −*y*+1, −*z*+1; (ii) *x*+1, *y*, *z*.
